# Identification of genes associated with reproductive function in dairy cattle

**DOI:** 10.21451/1984-3143-AR2018-0018

**Published:** 2018-08-03

**Authors:** M. Sofia Ortega

**Affiliations:** Division of Animal Sciences, University of Missouri, 65211, Columbia, MO, USA

**Keywords:** candidate genes, fertility, genomic selection, reproductive function.

## Abstract

The use of genomics has improved response to selection for functional traits with low heritability such as fertility traits. Much of the work on fertility traits has been performed through use of genome-wide association studies (GWAS) to identify genetic loci associated with reproductive traits. Under a GWAS approach, the assumption is that the markers on the panel are in linkage disequilibrium with causative mutations. In many cases, identification of the causative mutation is difficult because an associated genetic marker can be in intergenic regions and can be in linkage disequilibrium with variants in several nearby genes. Another approach is to identify candidate genes using knowledge of the biological pathways controlling a trait to search for single nucleotide polymorphism (SNP) in genes in those pathways. This should reveal putative causative markers responsible for genetic variation in biological function, and it is expected that the marker will be more strongly associated with a trait than one in linkage disequilibrium. An example of how a series of candidate gene studies demonstrate that identification of markers in genes involved in reproductive processes can lead to discovery of additional markers associated with genetic variation in reproductive traits is presented. In addition, the inclusion of candidate markers for fertility can improve reliability of genetic estimates for fertility traits, and the repeatability of the effects across a separate population of animals gives confidence that association elucidated by this set of markers is likely to be real. More importantly, the use of candidate genes can provide insights into the biology underpinning genetic variation in fertility, and that this understanding can lead to physiological interventions to improve reproductive function.

## Introduction

Fertility is a complex trait and, it is regulated in part by genetics. In the dairy cow, genetic merit for fertility and production are negatively correlated ranging from 0.35 - 0.60 (Boichard and Manfredi, 1994; VanRaden *et al*., 2004; Pritchard *et al*., 2013) and the intense selection for milk production during the last five decades has been one of the causes of a decrease in the genetic merit for fertility in dairy breeds (Butler, 2003). Nevertheless, improvements in reproductive performance of dairy cows has been made during the last decade because of advancements in reproductive management (Royal *et al*., 2000; Petersson *et al*., 2008), increased emphasis on genetic selection of reproductive traits (Norman *et al*., 2014), and incorporation of genomics into genetic selection schemes (García-Ruiz *et al*., 2016).

Most reproductive traits are controlled by many genes, each of which has a small effect. This is evident from genome wide association studies (GWAS) in which genetic variation in a trait is partitioned into associations with individual single nucleotide polymorphism (SNP). The low heritability characteristic of reproductive traits is indicative that only a small proportion of phenotypic variance is due to additive actions of individual genes and that reliability estimates of breeding values are prone to be low. It does not, however, mean that reproductive traits are not under genetic control, many specific genes have been identified that contain mutations that are associated with reproductive function. Furthermore, clear differences in fertility have been found between genetic lines of animals. In Holstein, for example, cows with higher genetic merit for fertility had fewer services per conception, and shorter intervals from calving to conception compared with cows with low genetic merit for fertility (Cummins *et al*., 2012a; Ortega *et al*., 2017a).

## Reproductive traits in dairy cattle

In the United States, three main female fertility traits are used in official genetic evaluations of dairy cattle: daughter pregnancy rate (DPR), cow conception rate (CCR) and heifer conception rate (HCR). DPR is defined as the percent of cows eligible for breeding that become pregnant in each 21-day period (i.e., over one estrous cycle). Conceptually, DPR is the product of estrous detection rate (the percent of cows in estrus that are detected in estrus) and pregnancy rate per insemination (the percent of inseminated cows that become pregnant). Practically, DPR is calculated from the term days open, which is the interval from calving to conception. Predicted transmitting ability (PTA) for DPR and days open are nearly linear function of each other. An increase of 1% in PTA for DPR equals a decrease of 4 days in the PTA for days open (VanRaden *et al*., 2003). Cow conception rate is defined as the percent of lactating cows that become pregnant after each service while HCR is the same variable for heifers (VanRaden *et al*., 2004). Heritability for these traits in Holsteins range from 0.001 - 0.016 (Pryce *et al*., 2004; VanRaden *et al*., 2004; Kuhn *et al*., 2006). Nonetheless, low heritability has not prevented progress in genetic selection for fertility. During the last 15 years, breeding values for DPR have improved, in part as a result of including fertility traits into economic indexes such as net merit and by inclusion of genomic information for breeding value calculations.

## Types of mutations responsible for genetic variation in reproduction

**Figure 1:**
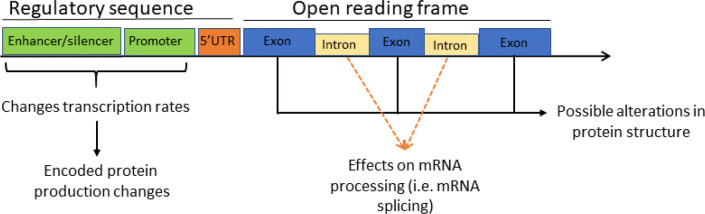
Possible effects of mutations relative to its location within a gene structure.

Genetic variation is the basis of biological diversity in a population. In the bovine genome, the total sequence length is 2,670,123,310 bp (UMD 3.1.1). As of December 2017, there were 102,499,615 SNP and 10,462 other genetic structural variations (>50 base pairs) including deletion/insertions, copy number variant, duplications, inversions, translocations and complex chromosomal arrangements (Aken *et al*., 2017). Genetic mutations ultimately affect the proteome of the organism either by affecting the structural properties of a protein or by modifying amount of protein in specific tissues. Most of the genetic studies are based in the association of SNP genotypes with a specific phenotype. How the physical location of a mutation relative to the coding and regulatory regions of specific genes can cause variation in phenotype is illustrated in Fig 1.

## Approaches for gene discovery and genetic selection for fertility

### Genome-wide association studies

GWAS are used to localize genomic regions that contribute to genetic variation of a trait. This approach is based on linkage disequilibrium, which refers to the association of any pair of alleles at different loci. Linkage disequilibrium exists because genes located closely together on a chromosome are more likely to be inherited together; i.e., cross-over events in meiosis are less likely to occur between two loci close together than two loci further apart or on different chromosomes. In a typical GWAS, thousands of SNP are interrogated for association with phenotypic variation in a trait, based on the assumption that SNP studied are in linkage disequilibrium with the causative mutation (Weller and Ron, 2011).

There are advantages and disadvantages of GWAS (Stringer *et al*., 2011; Riancho, 2012; Frąszczak and Szyda, 2016; Zondervan *et al*., 2016). The approach is unbiased with respect to previous knowledge of the trait of interest. Moreover, interrogation of a dense number of SNP across the genome can reveal novel markers associated with a trait. On the other hand, the large number of statistical tests performed during the analysis can lead to false positives so that stringent significance thresholds are necessary. One result is that markers with large effects are more likely to be detected and some important markers having a smaller effect may not reach significance. To get around these problems, large sample sizes are required to detect associations, particularly when multiple genes are involved in a trait. A limitation of GWAS is that often markers with significant associations with a trait are located in intergenic regions, and even when they are in linkage with the causative mutation, it is difficult to use this information to understand the basis of the genetic variance of the trait in question (Stringer *et al*., 2011; Riancho, 2012; Frąszczak and Szyda, 2016; Zondervan *et al*., 2016). Another limitation of GWAS is poor repeatability. Ioannidis *et al*. (2011) compiled results from a series of GWAS for human disease and found that replication of markers found by GWAS was around 1%. In cattle, the percent of significant SNP found in one population that were repeated in independent populations ranged from 0 (Littlejohn *et al*., 2012) to 18% (Höglund *et al*., 2014). Nevertheless, with appropriate sample sizes and statistical testing, GWAS can be very successful at identifying genes and genomic regions associated with specific traits. As an example, Cole *et al*. (2011) used a population of 1654 animals to identify 1586 SNP distributed in 486 genes that were associated with 31 production, reproduction, health and body conformation traits in Holstein cows. As more data becomes available, opportunities to validate these studies across populations become feasible, Liu *et al*. (2017) identified SNP in a Chinese Holstein population and those were later validated in a separate population of Nordic Holsteins. A detailed description of GWAS studies for female fertility can be found in the by Fortes *et al*. (2013), and the meta-assembly by Khatkar *et al*. (2014).

In dairy cattle, VanRaden *et al*. (2008) showed that incorporating data from GWAS into genetic estimates can improve reliability of genetic estimates over those based on parent averages. However, the amount of improvement depended on the trait, being higher for production traits (increases in reliabilities of 23-43%) than for DPR (17%; Wiggans *et al*., 2011). The lower increase for DPR probably reflects low heritability and the high degree of polygenicity. The inclusion of genomic information in dairy cattle improvement programs has been of particular importance in the artificial insemination industry, allowing more accurate selection of young bulls and increasing the rate of genetic gain (García-Ruiz *et al*., 2016). In Holstein cattle, during the last 7 years the inclusion of genomic information derived from GWAS has helped shorten generation interval in sires of bulls from 6.8 to ~2.5 years, boosted genetic gains for milk yield of from 50 to 109 kg per year, and for daughter pregnancy rate from negative values close to 0 to ~0.3 (García-Ruiz *et al*., 2016).

## Candidate gene approach

Another scheme to gene discovery is the use of candidate genes. A candidate gene is any gene thought to contain mutations responsible for a specific phenotype. Identification can be based on several approaches. The first is to search for genes located near genetic markers identified by GWAS. Kirkpatrick and Morris (2015) searched for candidate genes associated with ovulation rate in cattle. A GWAS was followed by Sanger sequencing of the target region in chromosome 10 which included *SMAD3, SMAD6* and *IQCH.* A total of 30 SNP in these genes were identified, and a haplotype comprising three SNP (two in *SMAD6* and one in *IQCH*) was associated with increased ovulation rate in daughters of bulls carrying the haplotype. After identification of a deficit of homozygotes for a JH1 haplotype associated with reduced fertility in Jersey using GWAS, sequencing performed in Jersey bulls revealed a nonsense mutation in *CWC15* which is embryonic lethal, as no homozygous individuals are present in the population (Sonstegard *et al*., 2013). Another approach was presented by Moore *et al*. (2016), where 58 candidate genes for regulation of fertility were identified by searching for genetic variants in differentially expressed genes in the endometrium and corpus luteum of cows with good or poor genetic merit for fertility.

Alternatively, candidate genes can be identified by using existing knowledge of the biological pathways controlling a trait, and search for SNP in genes in those pathways. Work at University of Wisconsin from the Khatib group has focused on using the candidate gene approach to identify genes associated with embryonic development. In one study, SNP were identified in eight genes in the POU1F1 pathway: *POUF1F1, GH, GHR, PRL, OPN, PRLR, STAT5A*, and *UTMP* (Khatib *et al*., 2009). There were significant associations for a SNP in *OPN* and *STAT5A* with fertilization rate, and for SNP in *GHR, STAT5A, PRLR* and *UTMP* with development of the embryo to the blastocyst stage. Likewise, Li *et al*. (2012), evaluated 25 genes of the TGFB signaling system. SNP were identified in *IBD3* associated with fertilization rate, and for a SNP in *BMP4* associated with development of the embryo to the blastocyst stage. Khatib *et al*. (2008a), studied the involvement of SNP in *FGF2* on embryonic survival because of the role of the FGF2 in regulation of *IFNT* expression in the trophectoderm (Michael *et al*., 2006). One SNP in the intron of *FGF2* was identified that was significantly associated with development of the embryo to the blastocyst stage (Khatib *et al*., 2008a). Likewise, and intronic SNP in *PGR* associated with fertilization rate and embryonic development to the blastocyst stage (Driver *et al*., 2009).

Tests of association for candidate genes have relatively high statistical power since the number of independent statistical tests is lower than for GWAS (Amos *et al*., 2011). Unlike GWAS, where genetic markers can change over time or between breeds because of crossover events during meiosis, the allelic association between a functional mutation and a genetically-controlled trait would be stable over time and more likely to extend across breeds. Furthermore, knowledge gained about the role of the gene in control of the trait could lead to improved understanding of the gene’s functionality (Zhu and Zhao, 2007; Weller and Ron, 2011). There are limitations to the candidate gene approach. First, it is not easy to determine whether the association of a SNP in a candidate gene is causative or is in linkage disequilibrium with a nearby functional SNP. Increased confidence that a SNP is causative if the same genetic variants have similar effects in an independent population. The best way to verify the functionality of a candidate SNP is often impractical for livestock, namely the use gene editing technology to produce animals with the mutation and evaluate effect on the phenotype of interest. Another problem with the candidate gene approach is that it is most useful for identifying causative mutations in the coding region of genes. However, much genetic variation is located outside the coding region - in the regulatory region of the gene and at distantly located loci involved in epigenetic regulation.

## Whole genome sequencing

Whole genome sequencing surveys the entire genetic code of an individual. The advantage of use whole genome sequencing is that it allows identification of complex forms of genetic variation besides SNP, including for example copy number variations. Moreover, by using whole sequencing the reliance on linkage disequilibrium disappears, as the causative mutation is on the generated data (Daetwyler *et al*., 2014). Haplotypes affecting fertility in dairy breeds previously identified with SNP50 chip (VanRaden *et al*., 2011), were further studied using whole genome sequence data by Fritz *et al*. (2013); and three novel mutations with damaged protein structure were identified in *GART, SHBG* and *SLC37A2* genes. Kadri *et al*. (2014), combining first SNP50 chip genotyping and whole genome sequencing identified a 660-kb deletion in chromosome 12 including four genes which is embryo-lethal in Nordic Red cattle. Using whole genome sequence data on 234 bulls, a mutation in *SMC2* was identified as causative for embryonic loss in cattle (Daetwyler *et al*., 2014). Given the rapidly decreasing cost of sequencing and the increase in number of animals in which whole genome sequences are available, it is likely that whole genome approaches to gene discovery are likely to predominate in the future.

## From genotype to function: a fertility story

Identification of genetic variants associated with reproduction can provide clues to understand fertility regulation. Cochran *et al*. (2013a), used a candidate gene approach to identify genes associated with genetic variation in female fertility in Holstein bulls. Genes were identified by searching the literature for two kinds of genes. The first were genes well known to be involved in reproductive processes such as steroidogenesis, follicular development and embryonic development. The second kind, were genes differentially expressed between various physiological conditions in tissues involved in reproductive function. Examples include genes differentially expressed in the endometrium of lactating vs non-lactating cows, and genes differentially expressed between embryos produced *in vitro* compared to embryos produced *in vivo*. In each candidate gene, SNP where identified and only those present in the coding region or regulatory region where selected.

The final list of SNP for analysis included 422 novel candidate SNP (1 SNP per gene) and 12 SNP previously associated with fertility in the literature including *CAST* (Garcia *et al*., 2006), *FGF2* (Khatib *et al*., 2010), *FSHR* (Yang *et al*., 2010), *GHR* (Waters *et al*., 2011), *HSPA1L* (Rosenkrans Jr. *et al*., 2010), *ITGB5* (Feugang *et al*., 2009), *LEP* (Brickell *et al*., 2010), *NLRP9* (Ponsuksili *et al*., 2006), *PAPPA2* (Luna- Nevarez *et al*., 2011), *PGR* (Driver *et al*., 2009), *SERPINA14* (Khatib *et al*., 2007), and *STAT5A* (Khatib *et al*., 2008b). A population of 550 Holstein bulls with divergent genetic merit for DPR, where bulls of low DPR were those with a PTA of -2 or lower, and bulls of high DPR had a PTA of +1.7 or higher was used to test association of SNP with fertility traits (DPR, CCR, and HCR). Significant association were found for 40 SNP with DPR, 22 with HCR, and 33 with CCR. The function of the genes associated with fertility included steroid biosynthesis, genes regulated by estradiol and progesterone and immune function. In a second study, the same SNP were tested in 93 bulls for association with sperm fertilization ability, and subsequent *in vitro* embryonic development (Cochran *et al*., 2013b). There were SNP in 12 genes associated with the percent of cleaved embryos that became blastocysts. From the genes containing SNP associated with percent of cleaved embryos that became blastocyst, *C1QB, MON1B, PARM1, PCCB, PMM2*, and *TBC1D24* were associated with DPR, *C1QB* and *PARM1* were associated with HCR, and *C1QB, MON1B, PARM1, PMM2, SLC18A2*, *TBC1D24* were associated with CCR.

More recently, SNP with significant associations with fertility found by Cochran *et al*. (2013a) were tested and validated in a separate population of Holstein cows with divergent genetic merit for fertility, cows were selected to have a high (≥1.5) or low PTA for DPR (≤-1.0). Of 51 genes previously associated with one or more estimates of fertility in bulls, 22 were associated with genotypic estimates of fertility in the cow population (Ortega *et al*., 2016a). In addition, SNP effects were associated with phenotypic measures of fertility, where animals carrying allelic variants associated with higher genetic merit for fertility also exhibited more favorable phenotypic measurements of fertility, having in general higher conception rates, fewer services per conception, and fewer days open (Ortega *et al*., 2017a). Thus, selection for those markers is likely to change actual reproductive performance. The list of SNP found associated with fertility in these studies can be found in Table 1.

There was a modest increase in reliability of genetic estimate for DPR (0.2%) when the SNP were included in the markers currently used for the national genetic evaluation system (Ortega *et al*., 2016a). This increase compares favorably to the 0.5% increase in reliability caused by adding up to 300,000 markers to the 50K bovine SNP chip (VanRaden *et al*., 2013). These findings indicate that the SNP under study here explain genetic variation not fully captured by GWAS and that the SNP are either causative or in higher linkage disequilibrium with the causal mutations than markers distributed across the genome.

The functions that were most represented by those genes containing SNP repeatedly associated with reproductive traits provides an indication of physiological processes important for variation among cows in reproductive function. There were 14 genes containing SNP associated with fertility that were regulated by estradiol and 6 by progesterone (Ortega *et al*., 2017a). Both steroids are essential for reproduction in mammals and there are compelling data indicating the importance of circulating concentrations of steroid hormones for cow fertility. Progesterone concentrations on days 4-7 after AI have been positively associated with pregnancy rate in Holstein heifers (Parr *et al*., 2012), and when follicular development occurs under low progesterone concentrations there is subsequent reduced fertility (Bisinotto *et al*., 2010). Circulating concentrations of steroids may be particularly important in high producing dairy cows, because steroid catabolism is increased and circulating concentrations of estradiol and progesterone are decreased (Wiltbank *et al*., 2006, 2014). It has been shown that cows with high genetic merit for fertility have larger corpora lutea and greater circulating concentrations of progesterone, and improved phenotypic fertility than cows with lower genetic merit for fertility (Cummins *et al*. 2012a, b; Moore *et al*., 2014).

The other function represented by genes with SNP associated with reproduction was immune function. Six genes associated with immune function were associated with genetic and phenotypic measures of fertility (Ortega *et al*., 2017a). Immune function is an important determinant of fertility. Cows that experience diseases postpartum have reduced reproductive function, are more likely to remain anovular, have decreased pregnancy rates and higher pregnancy losses than healthy cows (Santos *et al*., 2011, 2016; Ribeiro *et al*., 2016). In other studies, several of the genes differentially expressed in endometrium, liver, and muscle of Holstein cows with divergent genetic merit for fertility are involved in inflammatory processes (Moran *et al*., 2015, 2016). There is also evidence that cows can be identified by their immune response (high or low immune responders) and this is associated with the risk of developing diseases including retained placenta and metritis, which directly impact reproductive function (Thompson-Crispi *et al*., 2012).

Further research on the SNP in *COQ9* provided indirect evidence that function of the protein varied with genotype (Ortega *et al*., 2017b). *COQ9* was subjected to additional study because the SNP in this gene explained 3% of genetic variation in DPR in Holstein cows (Ortega *et al*., 2016a). COQ9 is involved in the biosynthesis of COQ10 (Tran and Clarke, 2007; Ben- Meir *et al*., 2015), which is a critical component of the mitochondrial electron transport system and which is required for mitochondrial ATP synthesis. The missense mutation studied causes a change in the predicted protein structure and was associated with a change in oxidative phosphorylation as reflected in changes in mitochondrial respiratory function. The allele associated with improved fertility was also associated with lower substrate requirements to maintain basal cellular function and reduced proton leaks from the electron transport system. *COQ9* is expressed in reproductive tissues, and these alterations could affect the function of these tissues by improving energy utilization of the cells. Additionally, because of reduced proton leak, the SNP could affect production of reactive oxygen species (Murphy, 2009; Jastroch *et al*., 2010). Further experimental work in the oocyte revealed that the variant associated with higher fertility was also associated with increased mitochondrial DNA copy number, which is associated with oocyte ATP production, successful oocyte maturation and fertilization (Reynier *et al*., 2001; May-Panloup *et al*., 2005; Tsai and St. John, 2016). Therefore, one of the reasons for differences in fertility among *COQ9* genotypes could reside in the allele associated with improved fertility, affects the competence of the oocyte due to higher mitochondrial content.

Another study was performed to understand the possible role of 12 genes containing SNP previously related to embryo competence to become a blastocyst by Cochran *et al*. (2013b). From the 12 genes, only two: *WBP1* and *PARM1* had increased expression at the moment of genome activation. Since the previous associations were based on the paternal SNP genotype, these were the genes most likely to represent actual effects of the SNP on embryonic development. Further evaluation showed that the SNP in *WBP1* caused changes in predicted protein structure. By reducing transcript abundance of this gene using Gapmer antisense oligonucleotides, it was revealed that *WBP1* plays a critical in trophectoderm formation. WBP1 is a single transmembrane adaptor protein (Pei and Grishin, 2012) that functions to bind a variety of signaling proteins containing the WW1 or WW2 domains. Among these are the proteins KIBRA, SAV1, and YAP involved in the Hippo signaling pathway (Zhao *et al*., 2010). Hippo signaling has been implicated in differentiation of the blastocyst in the mouse (Nishioka *et al*., 2009; Lorthongpanich *et al*., 2013). The transcription factor YAP interacts with TEAD4 to induce transcription of *CDX2* which in turn causes differentiation of the outer cells of the developing blastocyst into trophectoderm (Nishioka *et al*., 2009). Perhaps the effects of the SNP in *WBP1* modify the interactions of WBP1 with proteins of the hippo signaling pathway.

Evidence was also provided that the SNP in the promoter region of *HSPA1L* improves thermotolerance in the embryo (Ortega *et al*., 2016b). Previous work has associated this same mutation with increased calf crop in Brahman cattle (Rosenkrans Jr. *et al*., 2010), and with increased transcription of *HSPA1A/HSPA1L* (primers do not distinguish between the genes) in cells when exposed to high temperatures (Basiricò *et al*., 2011). Heat stress is known to affect fertility, particularly in dairy cattle, where cows in heat stress conditions show reduced pregnancy rates and pregnancies per AI (Gwazdauskas *et al*., 1973; Hansen and Aréchiga, 1999; Flamenbaum and Galon, 2010). In this study, expression of *HSPA1A/HSPA1L* was high at the 2-cell stage in the bovine embryo, and when putative zygotes were exposed to heat shock or high oxygen conditions, those embryos inheriting the deletion mutation in *HSPA1L* had greater survival after being exposed to adverse conditions. Perhaps embryonic survival during heat stress could be improved by selecting for thermotolerant genotypes.

Taking all together, this series of studies demonstrated that identification of SNP in genes involved in reproductive processes can lead to discovery of additional markers associated with genetic variation in reproductive traits. Inclusion of these markers in current genomic evaluations also can increase reliability of genetic estimates for fertility. The fact that SNP effects were frequently repeated among two independent populations of animals and that phenotype as well as genotype was affected provides confidence that selection of these markers will improve genetic merit for fertility. As shown for the SNP in *COQ9*, the use of candidate genes can provide insights of the biology underpinning genetic variation in fertility, and that this understanding can lead to physiological interventions to improve reproductive function.

**Table 1:** SNP associated with fertility traits in more than one candidate gene study^1^ .

Cow phenotype^2^					Cow genotype^2^				Bull genotype^3^			
SNP id	Gene	PR	SPC	DO		DPR	HCR	CCR		DPR	HCR	CCR
rs109967779	*ACAT2*					C		C		C		C
rs41766835	*APBB1*					G				G	G	G
rs133700190	*AP3B1*					T	T	T		T	T	T
rs109669573	*BCAS1*			C		C					C	
rs110217852	*BSP3*			A		A		A		A		
rs109332658	*C7H19orf60*					C		C		C		
rs135744058	*CACNA1D*						G			G	G	
rs137601357	*CAST*		T	T		T		T		T		T
rs109621328	*CD14*		C	C						C	C	
rs41711496	*CD40*						G	G		G		
rs133449166	*CSNK1E*					C	C			C	C	C
rs109137982	*FCER1G*	A	A	A		A						
rs43745234	*FSHR*	C									C	
rs41893756	*FUT1*		A	A		A		A		A		A
rs109262355	*FYB*		A	A							A	
rs109830880	*GCNT3*		T				T					
rs109711583	*HSD17B12*					G	G	G		G		
rs110828053	*HSD17B7*		C	C		C	C	C		C	C	C
rs110789098	*IBSP*	T					T	T				
rs111015912	*LDB3*		T							T	T	T
rs41256848	*LHCGR*		G			G						
rs134264563	*OCLN*		G	G		G		G		G		G
rs109813896	*PCCB*		C	C		C		C		C		
rs109629628	*PMM2*	G	G	G		G		G		G		G
rs133729105	*RABEP2*			G						G		G
rs110660625	*TBC1D24*	A	A	A		A				A		A

1 Shown are genes containing SNP in which a significant association between the SNP and one or more reproductive traits was observed in at least two studies. The letter represents the allele associated with superior reproduction. SNP significant in more than one study but where different alleles were associated with superior reproduction are not included in the table;

2 Based on the population of 2273 Holstein cows.

3 Based on a population of 550 Holstein bulls from Cochran *et al*. (2013a). The table is reproduced from the Journal of Dairy Science (Ortega *et al*., 2017a).

## Concluding remarks

The introduction of genomic selection in dairy cattle has increased rates of genetic gain, particularly for low heritability traits such as fertility. The use of GWAS as a tool for genomic selection has been very successful in improving accuracy of genetic selection in dairy cattle. The pathway to choose for gene discovery will depend on several variants: available information of the phenotype or trait of interest, population size and overall goal of the work. Without previous knowledge of genes involved in the phenotype of interest, GWAS are a powerful tool to identify regions associated with the trait. This also could elucidate candidate genes for further study as GWAS by themselves are not designed primarily to illuminate the underlying biology of the studied phenotype. The use of candidate genes in turn, allow also to improve the SNP panels used for genetic evaluations, by finding markers with stronger associations with the traits of interest that can be included in genomic evaluation schemes. Furthermore, with the identification of candidate genes, functional studies involving gene editing or gene knockout modifications can be developed to understand the tight regulation of reproductive function in cattle. As genotyping cost decrease, more datasets and whole genome sequence data becomes available that can be used to validate markers in different populations; and in the case of sequencing, identifying causal mutations of the phenotypes of interest.
